# State-dependent bioelectronic interface to control bladder function

**DOI:** 10.1038/s41598-020-79493-7

**Published:** 2021-01-11

**Authors:** James A. Hokanson, Christopher L. Langdale, Arun Sridhar, Phil Milliken, Warren M. Grill

**Affiliations:** 1 Department of Biomedical Engineering, Duke University, Durham, NC USA; 2 Department of Electrical and Computer Engineering, Duke University, Durham, NC USA; 3 Department of Neurobiology, Duke University, Durham, NC USA; 4 Department of Neurosurgery, Duke University, Durham, NC USA; 5 Galvani Bioelectronics, Stevenage, UK; 6 Department of Biomedical Engineering, Duke University, Fitzpatrick CIEMAS, Room 1139, Box 90281, Durham, NC 27708 USA

**Keywords:** Biomedical engineering, Urology, Neuroscience

## Abstract

Electrical stimulation therapies to promote bladder filling and prevent incontinence deliver continuous inhibitory stimulation, even during bladder emptying. However, continuous inhibitory stimulation that increases bladder capacity (BC) can reduce the efficiency of subsequent voiding (VE). Here we demonstrate that state-dependent stimulation, with different electrical stimulation parameters delivered during filling and emptying can increase both BC and VE relative to continuous stimulation in rats and cats of both sexes. We show that continuous 10 Hz pudendal nerve stimulation increased BC (120–180% of control) but decreased VE (12–71%, relative to control). In addition to increasing BC, state-dependent stimulation in both rats and cats increased VE (280–759% relative to continuous stimulation); motor bursting in cats increased VE beyond the control (no stimulation) condition (males: 323%; females: 161%). These results suggest that a bioelectronic bladder pacemaker can treat complex voiding disorders, including both incontinence and retention, which paradoxically are often present in the same individual.

## Introduction

The lower urinary tract, comprising the bladder, urethra, and external urethral sphincter, serves to store and, at an appropriate time and place, evacuate urine. Bladder dysfunction, including overactive bladder (OAB)—which includes urinary urgency or the sudden feeling of needing to urinate, urinary frequency, and urinary incontinence—as well as urinary retention, are highly prevalent conditions that lead to significant medical complications and decreased quality of life^1,2^. For those who are not responsive to conservative therapies, second line options include β3-adrenergic agonists and anticholinergics. However, these drugs have 1 year persistence rates of only 12–25% for anticholinergics and 32–38% for β3-agonists^3^. Third line therapies, including sacral neuromodulation (SNM) and posterior tibial nerve stimulation (PTNS), have success rates (≥ 50% reduction in symptoms) of 29–76% and 54–59% respectively^4^. In a randomized clinical trial of women with severe urgency incontinence (ROSETTA), an additional 3^rd^ line treatment option, OnabotulinumtoxinA (Botox) had a success rate (61%) similar to SNM (51%)^5^. However, Botox use can lead to urinary tract infections and/or urinary retention, which may require catheterization. In the ROSETTA study, complete resolution of symptoms occurred in only 20% of women with Botox and 4% of women with SNM. These data make clear that alternative therapies are needed for treatment of bladder dysfunction.

**Figure 1:**
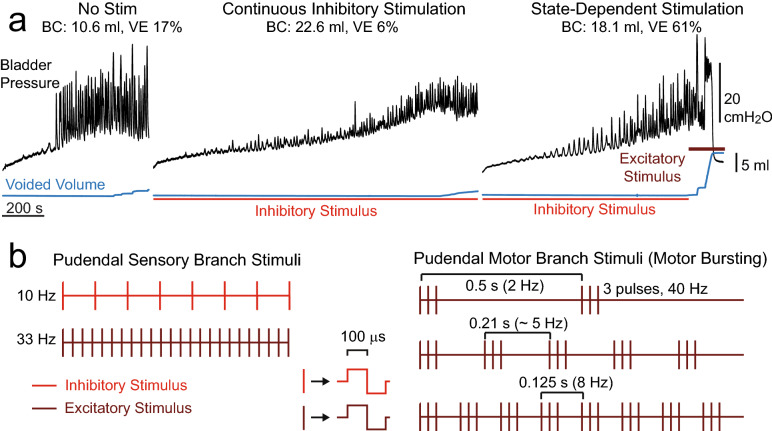
Patterns of state-dependent electrical stimulation to restore bladder function. (**a**) Example cystometrograms (male cat) showing bladder pressure (black) and voided volume (blue) during bladder filling and emptying without stimulation, during continuous stimulation to inhibit the bladder and promote continence, and during state-dependent stimulation to restore both continence and efficient bladder emptying. Bladder capacity (BC) and voiding efficiency (VE) for each trial are listed above the traces. (**b**) Stimuli were applied to the pudendal sensory branch (red) to inhibit the bladder and promote bladder storage. Stimuli were applied to either the pudendal motor branch (brown) or the pudendal sensory branch (33 Hz, cats only) to activate the bladder and promote bladder emptying. Excitatory stimuli, 33 Hz stimulation, 2 Hz motor bursting, and 5 Hz motor bursting are based on previous studies^9,10^ with an additional 8 Hz motor bursting pattern added for comparison.

Herein we demonstrate the function of a bioelectronic bladder pacemaker that uses selective electrical stimulation of peripheral pudendal nerve fibers to restore both urine storage (continence) and efficient evacuation (micturition or voiding). Electrical stimulation produces consistent increases in bladder storage capacity, to promote continence and potentially treat OAB^6^. However, strong inhibition of the bladder resulting from continuous sensory stimulation generates acute urinary retention, i.e., a substantial reduction in the efficiency of subsequent voiding^6–8^. These observations of stimulation-induced urinary retention inspired the concept of state-dependent stimulation: use of one set of electrical stimulation parameters to promote continence during the storage phase and another set of parameters to generate efficient emptying during the voiding phase (Fig. 1). This approach differs from traditional stimulation paradigms that use continuous stimulation regardless of bladder state. We demonstrate the utility of differential state-dependent stimulation, not only to increase bladder capacity (the volume the bladder can hold, BC) but also to increase voiding efficiency (the proportion of fluid expelled from the bladder during voiding, VE) in both rats and cats of both sexes.

## Results

In rats and cats of both sexes the impact of state-dependent stimulation relative to continuous stimulation and no-stimulation controls was assessed based on changes in BC and VE during single-fill cystometrograms. Continuous stimulation was always delivered at 10 Hz on the sensory pudendal nerve (rats, female cats) or dorsal genital nerve (DGN, a branch of the sensory pudendal nerve, male cats) throughout the entire cystometrogram. For state-dependent stimulation, 10 Hz stimulation was terminated just prior to voiding, and several approaches to promote efficient bladder emptying were tested: no stimulus during voiding (“fill only” condition); application of a bursting pattern on the pudendal motor branch (motor bursting); and 33 Hz stimulation of the pudendal sensory branch/DGN (cats only) (Fig. 1).

### State-dependent stimulation increased bladder capacity and voiding efficiency in rats

**Figure 2:**
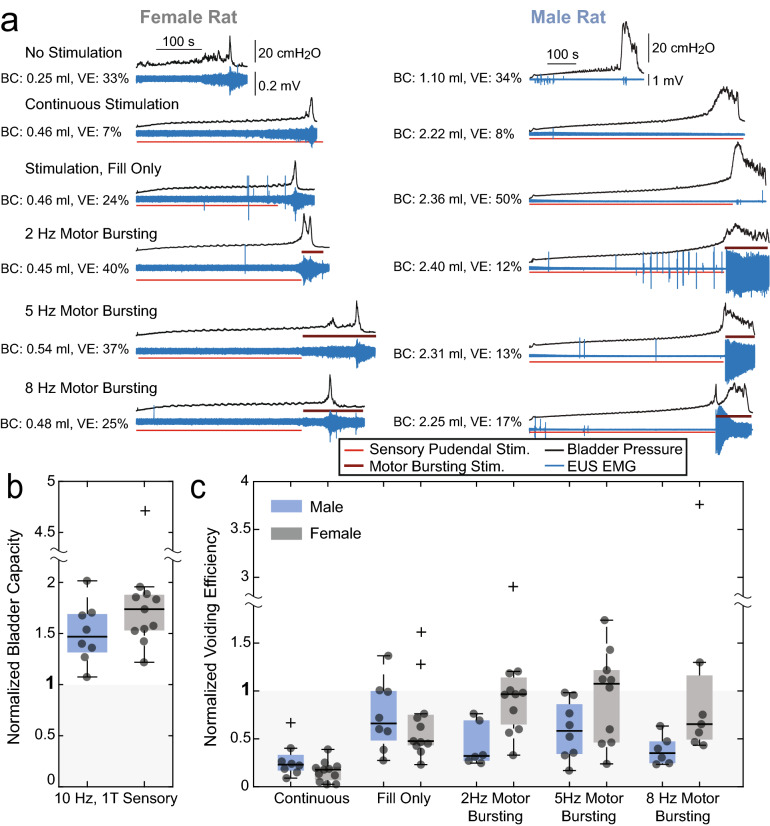
State-dependent stimulation restored both continence and efficient bladder emptying in male (n = 8) and female (n = 11) rats. (**a**) Cystometrograms from single experiments showing bladder pressure (black) and sphincter EMG (blue). Red lines indicate the duration of sensory pudendal stimulation (10 Hz, 1 T) to inhibit the bladder and promote storage. Brown lines above the traces indicate the duration of motor bursting stimulation (3 pulses at 40 Hz at the indicated burst repetition frequency) to activate the bladder and promote emptying. Average BC and VE for each type of trial for the example experiment are listed next to the traces. (**b**) BC for all experiments relative to controls. All relevant trials within an experiment were averaged resulting in a single data point for each experiment. Averages were 148% (males, P = 0.0008, n = 8) and 180% (females, P = 0.0002, n = 11). (**c**) VE for all stimulation conditions relative to controls. Continuous stimulation decreased VE relative to controls (males: 24%, P = 0.0006, n = 8; females: 12%, P < 0.0001, n = 11). State-dependent stimulation increased VE relative to continuous stimulation. In females motor-bursting stimulation increased VE beyond the stimulation during fill-only condition (2 Hz: P = 0.0049, n = 11; 5 Hz: P = 0.010, n = 10; 8 Hz: P = 0.0082, n = 7). In males, the largest increase in VE was the fill-only condition, increasing VE from 12 to 66% relative to control values (P = 0.011, n = 8). Box plots have a line at the median and the box extends from the estimated 25th to 75th percentiles. Lines beyond the box show the extent of data points that were not considered to be outliers (*boxplot* function, Matlab). Outliers are plotted as ‘+’ marks although all data were included for analysis. Paired *t* tests were used for BC comparisons. Mixed-effects analysis (i.e., an ANOVA like test) with two-stage linear step-up procedure of Benjamini, Krieger, and Yekutieli was used to determine the effect of condition on VE^11^.

Continuous stimulation of the pudendal sensory nerve at 10 Hz, 1 T (1 times threshold amplitude) reliably increased BC in both male and female rats (Fig. 2). Average BCs were 148% [126–174%] of control (mean [95% Confidence Interval], P = 0.0008, n = 8) in males and 180% [143–228%] of control (P = 0.0002, n = 11) in females. However, continued stimulation of the pudendal sensory nerve during voiding decreased VE to 24% [14–39%] of control in males (P = 0.0006, n = 8) and to 12% [6–22%] of control in females (P < 0.0001, n = 11).

VE was dependent on the stimulation condition during the voiding phase (males: P = 0.0017, n = 8; females: P < 0.0001, n = 11, mixed-effects analysis). Termination of pudendal sensory nerve stimulation at the onset of voiding (“fill-only” condition) increased VE from 24% with continuous stimulation to 58% [31–108%] of control in males (P = 0.011, n = 8, vs. continuous stimulation) and from 12% with continuous stimulation to 66% [42–103%] of control in females (P < 0.0001, n = 11, vs. continuous stimulation). In female rats initiation of pudendal motor nerve bursting at the onset of voiding further increased VE to 91% [63–131%] relative to control at 2 Hz (P = 0.0049, n = 11, vs. fill-only), 81% [52–125%] at 5 Hz (P = 0.010, n = 10, vs. fill-only), and 83% [44–157%] at 8 Hz (P = 0.0082, n = 7 vs. fill-only). However, in male rats pudendal motor nerve bursting at the onset of voiding did not increase VE; average VEs were the same or less than the 66% VE when pudendal sensory nerve stimulation was terminated at the onset of voiding (2 Hz: 39% [24–63%], P = 0.0841, n = 6; 5 Hz: 51% [31–85%], P = 0.1108, n = 8; 8 Hz: 36% [24–52%], P = 0.0120, n = 6; percentages relative to control, statistical comparisons vs. fill-only stimulation condition).

### State-dependent stimulation increased bladder capacity and voiding efficiency in cats

**Figure 3:**
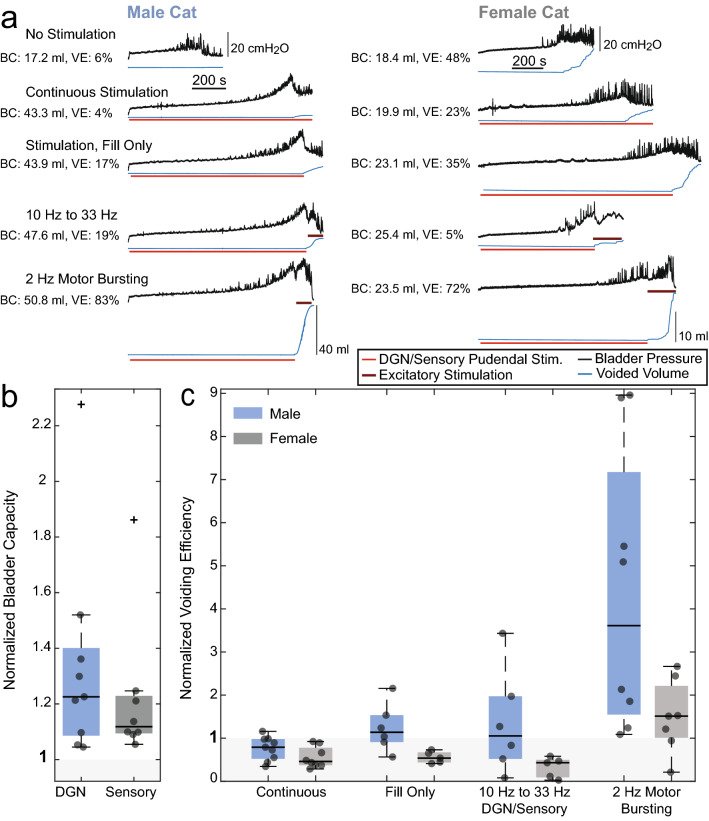
State-dependent stimulation restored both continence and efficient bladder emptying in male (n = 9) and female (n = 8) cats. (**a**) Cystometrograms from single trials with bladder pressure (black) and voided volume (blue) shown. Red lines indicate the duration of sensory pudendal stimulation (10 Hz, 1 T) to inhibit the bladder and promote storage. Brown lines above the voided volume traces indicate the duration of 33 Hz sensory stimulation or motor bursting stimulation to activate the bladder and promote emptying. Average BC and VE for each type of trial for the example experiments are listed next to the traces. (**b**) Normalized BC for all experiments relative to controls. All relevant trials within an experiment were averaged together resulting in a single data point for each experiment. Average normalized BCs were 130% (males, P = 0.011, n = 9) and 120% (females, P = 0.027, n = 8). (**c**) Normalized VE for all stimulation conditions relative to controls (males: P = 0.013; females: P = 0.022). Continuous stimulation decreased VE relative to controls (males: 71%, P = 0.028, n = 9; females: 51%, P = 0.013, n = 8). State-dependent stimulation with motor bursting increased not only BC but also VE in males (452%, P = 0.004, n = 8) but not females (235%, P = 0.069, n = 7) relative to continuous stimulation. In females, removal of one explainable outlier (Fig. 4) yielded an increase in VE relative to continuous stimulation (307%, P = 0.007, n = 6) and controls (161%, P = 0.032, n = 6). Paired *t* tests were used for BC calculations. Mixed-effects analysis with two-stage linear step-up procedure of Benjamini, Krieger, and Yekutieli was used for VE testing.

**Figure 4:**
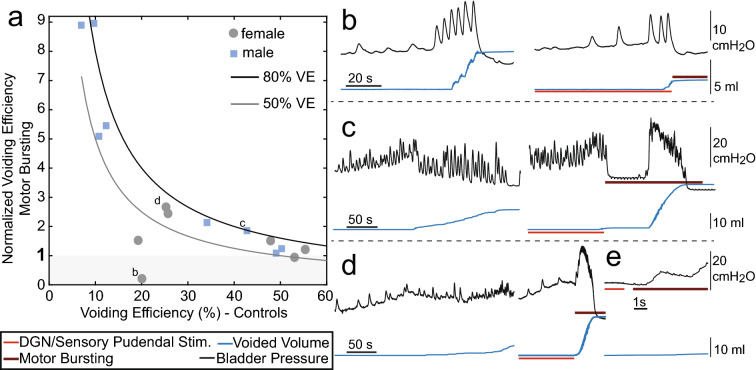
Factors influencing VE during motor bursting. (**a**) VE during motor bursting as a function of VE during control trials. Each point represents average data from one experiment. For reference, lines corresponding to 50% and 80% VE are shown (e.g., a value of 5 for normalized voiding efficiency at 10% control VE corresponds to 50% VE during stimulation). In one experiment (**b**) motor bursting was not effective due to short duration voiding contractions (outlier previously referenced in female VE results). (**b**–**d**) Cystometrograms from three different cats show control (non-stimulation, left) and state-dependent stimulation with motor bursting (right) with letters corresponding to labeled points in a. Trials are shown just prior to and during voiding. (**b**) Experiment with short duration bladder contractions. Motor bursting was started after the first epoch of urine expulsion, and the lack of subsequent urine expulsion rendered motor-bursting unable to increase VE. (**c**) Example where there was a long delay between the onset of motor bursting and start of the voiding contraction. (**d**) Example where there was a short delay between the onset of motor bursting and start of the voiding contraction. (**e**) Close-up of (d) showing that the contraction began approximately 1 s after the start of stimulation.

Continuous stimulation of the pudendal sensory nerve at 10 Hz, 1 T reliably increased BC in both male (130% [108–157%] of control, mean [95% CI], P = 0.011, n = 9) and female (120% [103–140%], P = 0.027, n = 8) cats, but also decreased VE to 71% [52–98%] of control in males (P = 0.028, n = 9) and 51% [36–73%] of control in females (P = 0.013, n = 8) (Fig. 3). VE was dependent on the stimulation condition during the voiding phase (males: P = 0.013, n = 9; females: P = 0.022, n = 8, mixed-effects analysis). Motor bursting was the most effective state-dependent approach for increasing VE in males, and increased VE both relative to continuous stimulation (452% [220–929%], P = 0.004, n = 8) and relative to control (323% [161–647%], P = 0.006, n = 8). In females, motor bursting was also the most effective state-dependent stimulation approach, and increased VE relative to continuous stimulation (307% [185–510%], P = 0.007) and relative to control (161% [107–241], P = 0.032, n = 6), when an explainable outlier was removed (see Fig. 4). However, the average changes of 235% [110–502%] relative to continuous stimulation and 120% [56–257%] relative to control were otherwise not significant (P = 0.069 and P = 0.60 respectively, n = 7).

Termination of pudendal sensory nerve stimulation at the onset of voiding increased VE in males relative to continuous stimulation (160% [92–277%], P = 0.044, n = 6), but not in females (106% [53–213%], P = 0.693, n = 5), and 33 Hz stimulation did not produce changes in VE in either sex relative to VE with continuous stimulation (males: 115% [41–320%], P = 0.328, n = 6; females: 40% [11–150], P = 0.177, n = 5).

The variance in the increases in VE generated by motor bursting prompted further investigation into factors influencing VE during motor bursting in cats. The VE during motor bursting was inversely dependent on the control VE (Fig. 4a). For control VEs near 50%, motor bursting reversed the reduction in VE resulting from prior inhibitory stimulation during filling, but did not increase VE beyond control values. However, for lower control VEs, motor bursting often increased VE by a factor of 2 or more. In one experiment where motor bursting was repeatedly initiated after the first voiding contraction, and sufficient time was not allowed for a second contraction, motor bursting was ineffective (Fig. 4b; data point of lowest VE with motor bursting in females in Fig. 3c). Additional examples of the timing of voiding bladder contractions relative to the onset of motor bursting are shown in Fig. 4c,d. Unlike in Fig. 4b, in Fig. 4c stimulation continued past the initial loss of urine and stimulation promoted emptying at the next bladder contraction, 63 s after stimulation onset. An example of more immediate voiding is shown in Fig. 4d. A closeup (Fig. 4e) shows that the increase in pressure came approximately 1 s after the onset of stimulation. Median times from onset of motor bursting to a bladder contraction were 0.63 s for males and 32.4 s for females. There were no apparent relationships between VE and the delay between motor bursting stimulation and the onset of the voiding contraction (r = 0.065, P = 0.82) or between VE and the duration of the contraction during motor bursting (r = 0.25, P = 0.38). These results indicate that motor bursting did not initiate bladder contraction, but rather amplified endogenously occurring bladder contractions to generate more efficient voiding. In both sexes motor bursting increased peak bladder pressure during voiding relative to controls (females: 140% [113–167%], P = 0.0086, n = 7, males: 125 [113–136%], P = 0.0023, n = 8).

**Figure 5:**
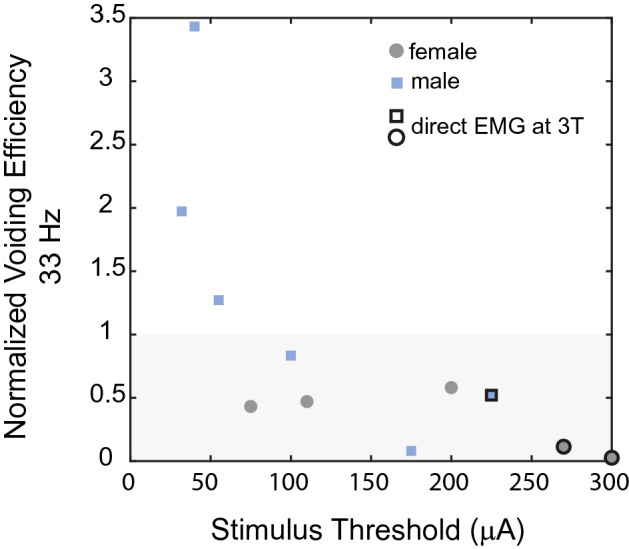
Changes in VE during 33 Hz stimulation were inversely related to the stimulation threshold to evoke reflex EMG activity. In some cases stimulation testing, which was always conducted at 3 times threshold (3 T), led to direct (< 1 ms latency) external anal sphincter (EAS) EMG activation (indicated by outlines around markers).

State-dependent stimulation that switched the frequency of sensory nerve stimulation from 10 to 33 Hz produced variable effects on VE in males, causing an increase in VE in 3/6 experiments, and in females VE during 33 Hz stimulation never exceeded control values. There was an inverse relationship between VE during 33 Hz stimulation and the stimulus amplitude necessary to evoke reflex EMG activity (Fig. 5), and in the three experiments with the lowest stimulation thresholds, VE exceeded control values.

### Mechanisms of action of motor bursting

**Figure 6:**
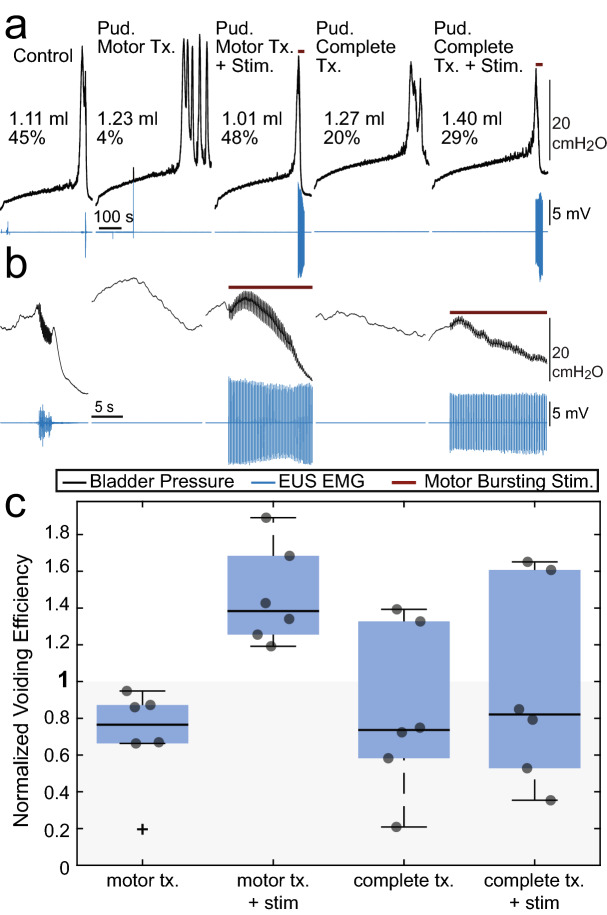
Motor-bursting stimulation in male rats after bilateral motor and sensory nerve transections. (**a**) Example cystometric traces from all five trial types with bladder pressure in black and EUS EMG in blue. Brown lines above the traces indicate the duration of motor bursting stimulation (3 pulses at 40 Hz at 5 Hz burst repetition frequency, efferent activation only due to transection). (**b**) Same trials as (**a**), but zoomed-in to show more clearly bladder pressure during voiding. (**c**) Average VE in each condition. Motor-bursting increased VE in all rats (P = 0.0082, n = 6, vs. controls; P = 0.013, n = 6, vs. motor transection, tx.). Following complete pudendal nerve transection motor-bursting failed to increase VE to the same level as when the sensory pudendal nerves were intact (P = 0.031, n = 6). Repeated measures one-way ANOVA P = 0.048. Follow-up testing conducted using the two-stage linear step-up procedure of Benjamini, Krieger, and Yekutieli.

Motor bursting in male rats did not generate an increase in VE over control. Therefore, we determined whether motor bursting increased VE after nerve transection (i.e., in the absence of endogenous motor bursting) and without a preceding inhibitory stimulus on the sensory branch (Fig. 6). Motor nerve transection did not reduce VE (63% [34–117%] relative to controls, P = 0.059, n = 6), however, only half of the six experiments demonstrated endogenous motor bursting. In those experiments VE was reduced to 51% (mean) of control values whereas in the other three experiments without endogenous bursting average VE was 89% of controls. In all experiments stimulation with motor bursting following motor nerve transection increased VE. VE increased to 145% [120–174%] of control (P = 0.0082, vs. controls, P = 0.013, vs. motor transection, n = 6). Subsequent bilateral sensory nerve transection (in addition to the bilateral motor nerve transection) eliminated the amplifying effects of motor bursting, and VE during motor bursting (83% [44–157%] relative to controls) was lower than with motor bursting prior to sensory nerve transection (58% [34–98%], P = 0.031, n = 6). These data indicate the effects of motor bursting are mediated by sensory feedback, secondary to the generated EUS contractions.

## Discussion

Continuous stimulation of afferents in the pudendal sensory nerve increased BC in rats and cats of both sexes. However, strong inhibition of the micturition reflex during filling led to profound decreases in the efficiency of subsequent voiding. State-dependent stimulation, implemented either as cessation of inhibitory sensory stimulation during voiding or switching to parameters intended to promote efficient voiding, enabled stimulation to increase both BC and VE. Importantly, these effects were observed across two species and both sexes and suggest that a bioelectronic bladder pacemaker can treat complex voiding disorders, which often involve, somewhat paradoxically, both incontinence and retention. For example, individuals have difficulty maintaining continence and efficiently emptying their bladder, i.e., detrusor hyperactivity with impaired contractility or DHIC^12^, and these persons may benefit from a state-dependent stimulation approach.

These results suggest that a bioelectronic bladder pacemaker can treat both incontinence and retention. In a clinical implementation the user could simply indicate manually that it was time to switch from a continence mode to a voiding mode to transition from the inhibitory stimulus to the excitatory bladder stimulus. The most efficient voiding resulted from stimulation of the motor branch of the pudendal nerve, while stimulation of the sensory branch inhibited the bladder to promote filling. Interfacing with both nerves could be accomplished with two separate distal leads^13^ or through fascicle-selective stimulation with a multi-contact electrodes placed more proximally on the compound pudendal nerve^14,15^.

The limitations of current therapies to treat incontinence^16,17^ indicate the need for innovative approaches that are more effective at maintaining continence. However, increases in the effectiveness of bladder inhibition and the accompanying increases in continence—potentially through the use of increased stimulation amplitudes^6^ or more effective timing of stimulation^18,19^—may also make it more difficult subsequently to expel urine, thus necessitating state-dependent stimulation.

The increases in VE generated by motor bursting were mediated by (mechanical) engagement of pudendal sensory afferents. Mechanical contraction of the urethra arising from motor bursting drives activation of urethral afferents to promote a sustained bladder contraction^10,20,21^, and transection of pudendal sensory branches prevented the increase in VE generated by motor bursting^10^. Mechanistic experiments in male rats (Fig. 6) confirmed that motor-bursting increased VE following bilateral pudendal motor branch transection, highlighting that the effects of motor bursting are mediated by sensory feedback, secondary to the EUS contractions generated by stimulation. This suggests that the lack of increased VE with motor-bursting during state-dependent stimulation in male rats (Fig. 2) was due to persistent carry-over inhibition from the immediately preceding sensory pudendal stimulation or adverse interactions between intrinsic and stimulation-generated motor burst activity.

In contrast to rats, cats, like humans, do not normally exhibit phasic bursting of the EUS during voiding, and yet motor bursting generated profound increases in VE in cats^10^. Motor bursting led to large amplitude contractions and high flow rates (e.g., Fig. 4c,d), signifying that motor bursting was effective at increasing VE. Bursting stimulation did not immediately initiate voiding bladder contractions, and the delay in observing an efficient bladder contraction suggests that the circuit must be in “voiding mode” rather than a storage mode^22^ for motor bursting to influence VE.

We observed inconsistent effects of 33 Hz afferent stimulation on VE in male cats, although previous work demonstrated that 33 Hz stimulation of pudendal afferents can increase VE in male cats^9,23^. VE during 33 Hz stimulation was inversely related to the stimulus threshold and/or stimulation intensity (3 times threshold). It is unclear to what extent the variation in stimulus threshold was due to electrode placement versus the physiological state of the animal (i.e., central excitability). Similarly, it is unclear if the inverse relationship between stimulus threshold and increase in VE was driven by differences in degree of reflex activation or whether stimulation at 3 times threshold led to current spread that directly activated urethral sphincter motor neurons. Additionally, all prior data were generated without a preceding inhibitory stimulus, and the impact of a preceding inhibitory stimulus was not tested in these experiments.

In female cats 33 Hz stimulation did not increase VE relative to controls. Unlike in male cats, where the cranial sensory nerve is separable from the dorsal nerve of the penis, the cranial sensory nerve and dorsal nerve of the clitoris in female cats are not separable in the ischiorectal fossa. As such, stimulation in females was conducted on both nerves together (i.e., on the pudendal sensory branch). However, previous work in males demonstrated that 33 Hz stimulation of the sensory branch increased VE^23^, suggesting that co-stimulation of both nerves (dorsal nerve of the clitoris, cranial sensory) should not influence the ability of 33 Hz to increase VE.

The impact of 33 Hz stimulation of the sensory pudendal nerve on VE in female cats has not been tested. 33 Hz stimulation of the pudendal nerve using a percutaneous needle^24^ or nerve cuff placed on the compound pudendal nerve^25^ generates bladder contractions in female cats, however VE was not measured in either study. Preceding 10 Hz stimulation of the sensory nerve during bladder filling may have reduced the effectiveness of subsequent 33 Hz stimulation during voiding. Additionally, stimulus thresholds were above the stimulus thresholds where 33 Hz stimulation was effective at increasing VE in male cats (Fig. 5). Thus, whatever mechanism prevented VE increases at higher stimulation amplitudes in male cats may have also done so in female cats.

Pudendal nerve stimulation is a promising alternative approach to regulating bladder function that has been tested pre-clinically^6^ and clinically^26^. Despite evidence that pudendal nerve stimulation may in some cases be superior to sacral nerve stimulation^27^ it is not used clinically. Inhibition of the bladder by pudendal nerve stimulation during saline cystometry is mediated by a spinal GABAergic mechanism^28^ as well as by central nervous system glutamate receptors^29^ Despite this mechanistic understanding it is unclear how to determine whether patients will respond better to pudendal nerve stimulation or sacral nerve stimulation, and it remains to be determined whether the state-dependent stimulation approach will work with sacral nerve stimulation. Finally, this work was conducted in anesthetized quadrupeds and there may be important differences in humans response to this approach. Although cats, like humans, relax the urethral sphincter when voiding, pelvic floor muscles differ in quadrupeds and humans^30^. In previous experiments we noted that there appeared to be a slowly escalating bladder capacity over time which we hypothesized was stimulus driven^6^. A similar phenomenon was observed in the present experiments, as well. Average Pearson correlation coefficients (calculated per experiment then averaged) between trial number and bladder capacity were 0.41 [0.18–0.64] ([95% CI], female rats), 0.54 [0.35–0.73] (male rats), 0.60 [0.26–0.95] (female cats), and 0.46 [0.12–0.79] (male cats). These correlations were significant in 4/11 female rats, 4/8 male rats, 6/8 female cats, and 6/9 male cats. To assess how this change in control bladder capacity impacted the results, we computed changes in bladder capacity using sequential control trial-stimulation trial pairs, rather than averaging over all control trials and all stimulation trials. Changes in average bladder capacities and 95% confidence-intervals, relative to comparisons between averaged trails, were relatively minor, changing from 180% [143–228%] (mean [95% CI], stimulation values relative to controls, P = 0.0002) in our original analyses to 198% [154–254%] (P = 0.00013) in female rats, from 148% [126–174%] (P = 0.0008) to 146% [123–174%] (P = 0.0014) in male rats, from 120% [103–140%] (P = 0.027) to 115% [99–132%] (P = 0.0606) in female cats, and from 130% [108–157%] (P = 0.011) to 126% [109–145%] (P = 0.0065) in male cats. It is unclear over what duration these slow increases in bladder capacity persist and whether they contribute to the long-term effects observed with stimulation paradigms such as maximal electrical stimulation^31^ or tibial nerve stimulation^32^.

In summary, continuous stimulation of pudendal afferents increased BC but decreased VE in rats and cats of both sexes. In all models at least one form of state-dependent stimulation increased BC and VE beyond the VE observed during continuous stimulation. In male rats simply terminating the inhibitory stimulus during voiding was effective at increasing VE. In female rats, and male and female cats, the most effective stimulus at increasing VE was stimulation of the motor pudendal branch in a bursting pattern.

## Methods

All animal care and experimental procedures were reviewed and approved by the Duke University Institutional Animal Care and Use Committee. All experiments were performed in accordance with relevant guidelines and regulations.

### Rat surgical preparation and procedures

Acute experiments were conducted in male Wistar rats (n = 19) weighing between 396 and 541 g (median: 477 g) and female Wistar rats (n = 11, Charles River Inc.) weighing between 233 and 294 g (median: 245 g) anesthetized with urethane (1.2 g/kg SC, supplemented as necessary). Body temperature was monitored using an esophageal temperature probe and maintained at 36–38 °C with a water blanket. Heart rate and arterial blood oxygen saturation levels were monitored using a pulse oximeter (Nonin Medical Inc., 2500A VET).

#### Catheter and EMG electrode placement

Methods are similar to those reported previously^6^. The bladder was exposed via a midline abdominal incision. The tip of a polyethylene (PE-90) catheter (Clay Adams**,** Parsippany, N.J) was heated to create a collar and inserted into the bladder lumen through a small incision in the apex of the bladder dome and secured with 6-0 silk suture. The abdominal wall was closed in layers with 3-0 silk suture. The bladder catheter was connected via a 3-way stopcock to an infusion pump (Harvard Apparatus PHD 4400) and to a pressure transducer (ArgoTrans, Argon Medical Devices Inc., Plano, TX) connected to a bridge amplifier and filter (100 Hz low-pass, model: 13-6615-50, Gould Instruments, Valley View, OH) for measuring intravesical pressure. Pressure data were sampled at 1 kHz using a PowerLab system (AD Instruments, Colorado Springs, CO).

External urethral sphincter (EUS) EMG was measured using a bipolar paddle electrode (Microleads, Boston) placed intra-abdominally between the urethra and the pubic symphysis^33^. EUS EMG leads were connected through a preamplifier (HIP5, Grass Products, Warwick, RI) to an amplifier (P511, Grass Products). A subcutaneous needle served as ground. Signals were filtered (3 Hz–3 kHz) and sampled at 20 kHz.

#### Neural interface placement

After placing the bladder catheter and EUS EMG electrodes, the animal was turned to a prone position for placement of peripheral neural interfaces. A skin incision was made down the midline of the lower back and the gluteal muscles were resected from the midline. The sensory branch of the pudendal nerve in both sexes, as well as the motor branch of the pudendal nerve in females were accessed in the ischiorectal fossa. The ischium was spread apart from the sacrum to expose the fossa. The nerves of interest were dissected from other tissue with fine forceps, and bipolar circumneural cuff electrodes (CorTec, Freiburg, Germany) were placed. Nerve cuff sizes used were 200-2 (inner diameter in µm, length in mm) for the pudendal sensory in females, 400-3 for the pudendal sensory in males, and 400-3 for the pudendal motor in females. Due to the small size of the motor nerve branch in females, the cuff was placed around the pudendal motor branch as well as the pudendal blood vessels. This approach stabilized the interface and ensured small nerve branches were not missed. In females, due to space constraints, the two nerve cuffs (sensory, motor) were placed on opposite sides of the body.

**Figure 7:**
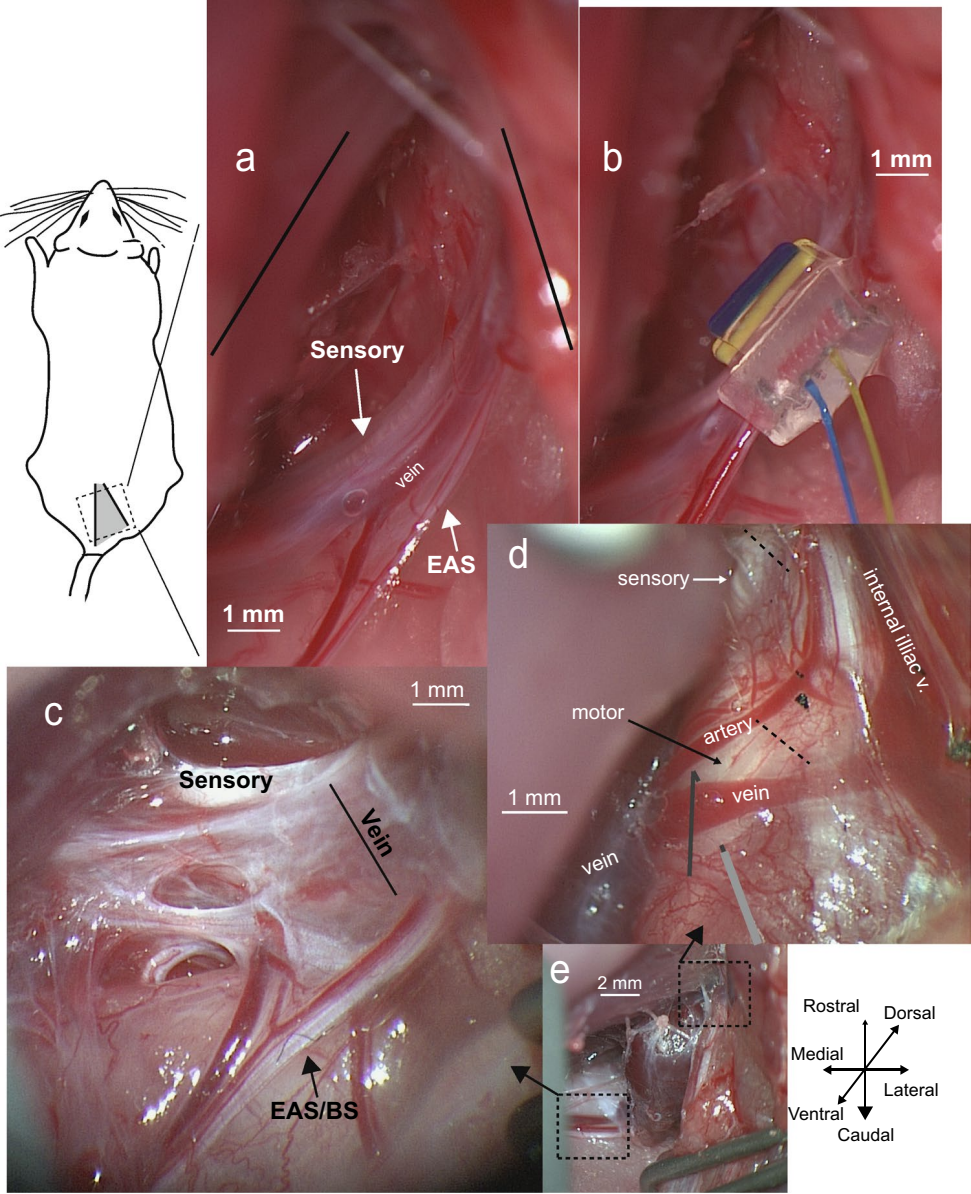
Rat pudendal anatomy and instrumentation. (**a**) Picture of the pudendal nerve branches in the ischiorectal fossa in a female rat. Nervous tissue innervating the urethral sphincter is spread between the internal pudendal vein and external anal sphincter (EAS) nerve (females) or external anal sphincter/bulbospongiosus (EAS/BS) in males see also: Ref.^34^, Figure 1. (**b**) Placement of a nerve cuff around the motor nerves (EUS and EAS branches) and blood vessels but not the pudendal sensory nerve (same animal as (**a**). (**c**) Picture of the pudendal nerve branches in the ischiorectal fossa in a male rat (some dissection is noticeable on the left side but was not done in the included experiments). Of note, the internal pudendal vein is much larger than in the female, making it difficult to place a nerve cuff around all motor neuron tissue and the blood vessel (as was done in female rats). (**d**) Picture in another male rat of the pudendal nerve branches on the dorsal surface of the ischium. Dotted lines mark points where the nerves were transected for the mechanism studies. In some animals, including this one, the motor branch appeared as two separate fascicles (side by side) that appeared to merge as the nerves went under the pudendal vein and into the ischiorectal fossa. The lines at the bottom center of the image represents where the stimulation wire was placed. The exposed tip of the wire was placed underneath the nerve with the tip bent back over the top then covered in Kwik-Cast. (**e**) Picture from another male rat to help with spatial orientation showing both the ischiorectal fossa (bottom-left, ventral) and dorsal surface of the ischium (upper-right). The pudendal vein swells considerably after leaving the dorsal surface of the ischium.

In male rats the pudendal sensory nerve cuff and a wire electrode on the pudendal motor nerve were placed ipsilaterally. The pudendal motor branch was accessed at the dorsal surface of the ischium, prior to the entry of the nerve into the fossa. Four strand stainless-steel wire (AS644, Cooner Wire Company, Chatsworth, CA) was placed around the pudendal motor nerve, as well as the internal pudendal vein and occasionally the pudendal artery, depending on space available. The wire was covered with Kwik-Cast (World Precision Instruments, Sarasota, FL) and a needle (22G × 1″, Model 511055, BD, Franklin Lakes, NJ) was placed subcutaneously on the side of the rat to serve as a return. In one set of experiments in males (mechanism studies), only the pudendal motor wire was placed. Example setups for both male and female rats are shown in Fig. 7.

#### Electrical stimulation

Electrical stimulation was delivered using a stimulus isolator (n = 10, model 220, A-M Systems, Sequim, WA) or a stimulus generator (n = 20, model STG4002-16 mA, MultiChannel Systems, Reutlingen, Germany). Stimulation pulses were charge-balanced biphasic symmetric rectangular pulses with 100 µs phase duration and no interphase gap. Strength of stimulation was assessed by monitoring evoked EUS EMG. Amplitudes for sensory pudendal nerve stimulation were normalized to the minimum stimulation amplitude necessary to evoke reflex EUS EMG activity (~ 25 ms latency), referred to as 1 T (1 times threshold amplitude). Motor branch stimulation was at the minimum stimulus amplitude required to evoke a maximal (direct) EUS EMG response.

Electrical stimulation of the sensory pudendal nerve to promote bladder storage (increase BC) was at 10 Hz and 1 T and started at the onset of bladder filling. Depending on the type of trial, stimulation either continued throughout bladder emptying or terminated just prior to bladder emptying. Electrical stimulation to promote bladder emptying started just prior to bladder voiding or urine leakage and continued throughout the bladder contraction. Four approaches to promote bladder emptying were tested. For the first approach no stimulation occurred, but rather the inhibitory stimulus was terminated such that no stimulation was delivered during bladder emptying. The other three approaches were burst patterns applied to the pudendal motor nerve consisting of three pulses at 40 Hz repeated at either 2 Hz, 4.76 Hz (herein referred to as 5 Hz), or 8 Hz^10^. In another set of experiments motor bursting at 5 Hz was delivered without a preceding inhibitory stimulus on the pudendal sensory branch.

#### Cystometric testing

After surgery the bladder was filled continuously with physiological saline at room temperature for at least 45 min. The pump rate was adjusted periodically so that voiding occurred once approximately every 4 min. The bladder was subsequently emptied and single-fill cystometrograms were conducted. The pump rate was set (males: 6–25 ml/h, 13 ml/h median; females: 2–8 ml/h, 5 ml/h median) so that non-stimulation trials took 7–10 min from the start of filling until voiding occurred. The pump rate was maintained throughout blocks of stimulation and non-stimulation trials. Each trial consisted of a period of no bladder filling or stimulation (quiet period) lasting 1–3 min in duration, followed by filling, and the pump was stopped once a void or urine leaking occurred. When the bladder contraction ceased the bladder was manually emptied via aspiration through the filling line, and the residual volume was recorded. If the bladder remained contracted for an extended period of time (1 or 1.5 min, consistent within experiment), the trial was stopped and the bladder was emptied. Voided volume was also collected and logged for each trial.

Trials of continuous inhibitory stimulation occurred after 2–4 non-stimulation (control) trials. The increase in BC from continuous stimulation was used to determine a pump-volume and corresponding bladder pressure (at that volume) that was suitable as a stopping point for subsequent state-dependent stimulation (i.e., the onset of motor bursting just prior to voiding or leaking). Whichever threshold (volume or pressure) that was reached first resulted in stimulation switching. The four types of state-dependent stimulation trials then followed in a randomized order, defining one block, and one to three blocks were collected per experiment.

In a second set of experiments, the mechanism of action of motor bursting was investigated in male rats, complementing previous work done in female rats and male cats^10^. In this second set of experiments non-stimulation control trials were followed by bilateral motor pudendal nerve transection in such a way that ensured efferent only transmission from electrical stimulation (Fig. 7d). This was followed by 30 min of continuous bladder filling. No-stimulation trials were conducted to establish a baseline, followed by interleaved no-stimulation and 5 Hz motor bursting trials, with at least two stimulation trials collected. This process—continuous bladder filling, stimulation and no-stimulation trials—was repeated again after bilateral pudendal sensory transection to assess the impact of lack of pudendal sensory feedback on the effects on bladder emptying generated by motor bursting.

#### Data analysis

Data were excluded from 5 male rats from mechanism testing experiments. In one rat the stimulation wire became dislodged following motor branch transection. The second excluded rat had bloody urine during initial cystometric testing. In another rat the bladder was unable to be emptied via aspiration. In another rat, reflex contractions were lacking, as, presumably too much supplemental urethane was administered. Finally, in the fifth excluded rat bilateral sensory transection led to a large increase in VE that was not typical of other experiments. At the end of that experiment an unusually large seminal plug was found to be covering the urethral meatus.

BC (the sum of the voided and residual volumes) and VE (voided volume divided by the BC) were calculated for each trial. Voiding efficiencies were compared between no-stimulation controls, continuous stimulation, and the state-dependent stimulation conditions using mixed-effects analysis, after log-transforming the normalized data, followed by the Benjamini, Krieger and Yekutieli two-stage step-up method for post hoc comparisons^11^ with the false discovery rate set at 0.05 (GraphPad, Version 8.2.1, La Jolla, CA). As the same stimulation parameters were used during the filling phase in all conditions, bladder capacities from all stimulation trials were averaged. For BC, normalized capacities were log-transformed and compared to controls via a two-tailed paired *t* test (GraphPad). Means and confidence intervals were calculated on the log-transformed data and then inverse-transformed to aid interpretation. A similar statistical approach was used for the mechanism experiments in male rats, however a repeated measures one-way ANOVA was used instead of mixed-effects analysis (no missing data present, GraphPad). Statistical significance is reported as P < 0.05.

Box-plots in figures were created using Matlab (2018b, Mathworks, Natick, MA). Box-plots are for data visualization only and were not used for statistical calculations or to exclude data.

### Cat surgical preparation and procedures

Acute experiments were conducted in adult neurologically intact male (n = 13, 3.1–4.1 kg, median: 3.5 kg) and female (n = 12, 2.2–3.8, median: 3.1 kg) cats (Liberty Research, New York). Methods are similar to those reported previously^35^. Anesthesia was induced with isoflurane (3%) and maintained with α-chloralose (65 mg/kg initial dose followed by continuous infusion of 5 mg/kg/h IV and supplemented as necessary based on jaw tone and blood pressure) following completion of surgery. Gentamicin 5 mg IM and ketofen 1.2 g/kg SQ were given prior to surgical incision. A tracheotomy was performed to place a silicone endotube (Cat. no J0612B, Jorgensen Laboratories, Loveland, CO) connected to an artificial respirator (ADS 1000, Engler Engineering Corporation, Hialeah, FL), and artificial respiration was controlled to maintain end-tidal CO_2_ between 3–4% (Capnogaurd, Novametrix Medical Systems Inc., Wallingford, CT). The right carotid artery was cannulated with a 3.5 Fr polypropylene catheter (Cat. no 8890703211, Medtronic, Minneapolis, MN) to monitor arterial blood pressure (Tektronix 413A Neonatal Monitor) and was kept patent by infusing saline at a constant rate of 6 ml/h. Body temperature was measured using an esophageal temperature probe and maintained at 38° C with a forced-air warming blanket (Bair Hugger model 505, 3 M). Additional fluids (0.9% physiological saline with 5% dextrose and 8.4 g/l NaHCO_3_) were administered continuously (15 ml/kg/h IV) via a catheter in the left cephalic vein.

#### Catheter and EMG electrode placement

Methods are similar to those reported previously^36^. Following a midline abdominal incision, the bladder was cannulated through the dome with a modified 14G BD Angiocath catheter connected to PE 90 tubing introduced with a hypodermic needle, secured with a purse string suture (4-0 silk, Cat. No M-S418R19, AD Surgical, Sunnyvale, CA), and connected to a solid-state pressure transducer (Deltran, Utah Medical, UT) to measure bladder pressure. A force transducer (model: MLT500D, AD Instruments, Colorado Springs, CO) was used to collect voided volume (VV). The external anal sphincter (EAS) EMG activity was measured by PFA-coated platinum-iridium wires (0.0055 inch-diameter, A-M Systems, Sequim, WA) inserted percutaneously into the EAS bilaterally. EAS EMG leads were connected through a preamplifier (HIP5, Grass Products, Warwick, RI) to an amplifier (P511, Grass Products). Bladder pressure (BP), VV, and EAS EMG signals were amplified, filtered, and sampled at either 1000 Hz (BP and VV) or 20,000 Hz (EAS EMG).

#### Neural interface placement

**Figure 8:**
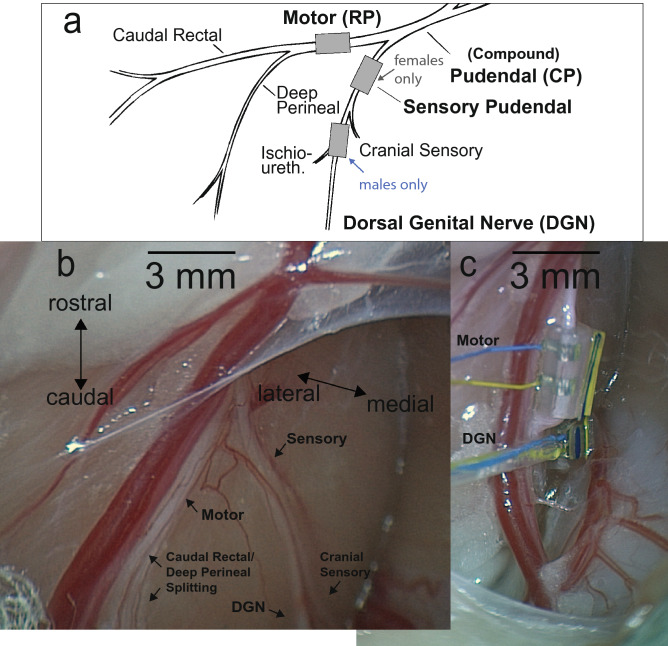
Cat anatomy and nerve cuff placement. (**a**) Neuroanatomy of the male cat lower urinary tract modified from^37^. In females a nerve cuff was placed on the sensory pudendal branch, as it is not possible with this (dorsal) approach to separate the cranial sensory nerve from the dorsal genital nerve. In males the dorsal genital nerve and ischiourethralis branches were isolated from the cranial sensory nerve. We refer to this stimulation site as stimulation of the dorsal genital nerve (DGN) or dorsal nerve of the penis (DNP), even though it also incorporates the ischiourethralis branch. (**b**) Photo of the pudendal nerve branches in a male cat. (**c**) Photo in the same cat after cuffs have been placed and retractors removed.

After bladder catheter placement the animal was rotated into the prone position. An incision was made near the base of the tail on the left side. Muscle and fat were resected, and the tissue was spread with retractors to access the ischiorectal fossa. In males the dorsal genital nerve was isolated from the cranial sensory nerve and a bipolar nerve cuff was placed around the isolated dorsal genital nerve (Fig. 8). In females a nerve cuff was placed around the sensory nerve (includes cranial sensory and dorsal genital nerve branches). In both males and females a nerve cuff was placed around the pudendal motor branch (a.k.a. the rectal perineal branch). A mix of 300-2 (inner diameter in µm, length in mm) and 400-3 nerve cuffs were used (CorTec, Freiburg, Germany), with the smaller diameter cuffs used primarily on the sensory nerve and the larger diameter cuffs primarily on the motor nerve (Fig. 8c). After nerve cuff placement the retractors were removed and the incision was covered with wet gauze and plastic wrap.

#### Electrical stimulation

Electrical stimulation was delivered using a stimulus generator (model STG4002-16 mA, n = 8 and model STG4004-16 mA, n = 17, MultiChannel Systems) using the same 100 µs per phase biphasic waveform used in rats. Strength of stimulation was assessed by monitoring evoked EAS EMG. For stimulation of the sensory pudendal nerve (females) or dorsal genital nerve (males) stimulus threshold was defined as the amplitude necessary to evoke reflex EAS EMG activity (~ 15 ms latency). For stimulation of the motor branch (i.e., rectal-perineal nerve) the minimum amplitude that evoked a maximal EAS EMG response was used.

Electrical stimulation of sensory pudendal nerve (female) or dorsal genital nerve (males) during the filling phase was at 3 T and 10 Hz^28^ and started at filling onset. Depending on the type of trial, stimulation either continued throughout bladder emptying or terminated just prior to bladder emptying. Electrical stimulation to promote bladder emptying started just prior to bladder voiding or urine leakage and continued throughout the bladder contraction. Three approaches to promote bladder emptying were tested. One approach was to terminate the stimulus at void onset. A second approach was to change the stimulation rate on the sensory pudendal or dorsal genital nerve from 10 to 33 Hz at void onset, still at 3 T, as 33 Hz stimulation was shown to increase VE in male cats^23^. The third approach was to deliver a bursting pattern to the motor branch of the pudendal nerve at void onset. All bursting patterns consisted of 3 pulses at 40 Hz at a 2 Hz train rate.

#### Cystometric testing

The bladder was filled continuously with physiological saline at room temperature using an infusion pump (model: PHD 4400, Harvard Apparatus), with an open urethra for approximately 1 h to allow post-surgical recovery. The bladder was subsequently emptied and cystometrograms recorded with the animal in a prone position. The pump rate was adjusted so that non-stimulation trials lasted for 15–20 min, with a fixed pump rate used per block (males: 0.6–4 ml/min, 2 ml/min median; females: 0.3–1.6 ml/min, 0.8 ml/min median). For each cystometrogram, the bladder was filled until voiding or leakage occurred, at which time the infusion pump was turned off. In six experiments (three male, three female) the bladder was emptied after bladder pressure returned to baseline. In the remaining experiments the time to emptying the bladder was fixed at three minutes after initial fluid loss. The bladder was emptied via the catheter using a syringe. Voided and residual volumes were recorded and used to calculate BC and VE. Trial order was as described for the rat experiments and 1 or 2 blocks were collected per experiment.

### Data analysis

Data were excluded from 8 cats. Three female cats were excluded due to a non-reflexive bladder after the initial dose of alpha-chloralose. One of these cats was given too much alpha-chloralose and never recovered. The other two were siblings and appeared to be sensitive to alpha-chloralose. In one male cat that showed no response to motor-bursting, post-mortem dissection revealed that the nerve cuffs were placed on the urethral (deep perineal) and anal (caudal rectal) branches of the motor nerve, rather than on the sensory and motor branches. Subsequent analysis of reflex data indicated that this likely had occurred in two previous male cat experiments which were also excluded from analysis. One female cat was excluded due to urine leakage during filling following the failure to washout intravesical Prostaglandin E2 from a previous experiment in the same animal. One female cat was not able to be effectively ventilated, CO_2_ values increased to 5.8% (target range 3–4%) and could not be reduced, and this cat was also excluded from analysis.

In all experiments where stimulus conditions are not present, those conditions were not tested (i.e., all data are missing at random). In two cat experiments (one male, one female), motor-bursting was planned but was not tested because the nerve cuff that had been placed around pudendal motor branch had become detached from the nerve.

The analysis of data from cats was the same as described for data from rats, except that differences in peak pressure during voiding were also examined. For each trial the maximum pressure during voiding (when urine was being expelled) was recorded. Peak values were compared in the same manner as BC and VE.
